# Development and validation of a prediction model on spontaneous preterm birth in twin pregnancy: a retrospective cohort study

**DOI:** 10.1186/s12978-023-01728-3

**Published:** 2023-12-21

**Authors:** Xiaofeng Yang, Qimei Zhong, Li Li, Ya Chen, Chunyan Tang, Ting Liu, Shujuan Luo, Jing Xiong, Lan Wang

**Affiliations:** 1 Department of Obstetrics and Gynecology, Chongqing Health Center for Women and Children, No.120 Longshan Road, Yubei District, Chongqing, 401147 China; 2 Department of Obstetrics and Gynecology, Women and Children’s Hospital of Chongqing Medical University, No.120 Longshan Road, Yubei District, Chongqing, 401147 China

**Keywords:** Spontaneous preterm birth, Prediction, Nomogram, Area under the curve

## Abstract

**Background:**

This study was conducted to develop and validate an individualized prediction model for spontaneous preterm birth (sPTB) in twin pregnancies.

**Methods:**

This a retrospective cohort study included 3845 patients who gave birth at the Chongqing Maternal and Child Health Hospital from January 2017 to December 2022. Both univariable and multivariable logistic regression analyses were performed to find factors associated with sPTB. The associations were estimated using the odds ratio (OR) and the 95% confidence interval (CI). Model performance was estimated using sensitivity, specificity, accuracy, area under the receiver operating characteristic curve (AUC) and decision curve analysis (DCA).

**Results:**

A total of 1313 and 564 cases were included in the training and testing sets, respectively. In the training set, univariate and multivariate logistic regression analysis indicated that age ≥ 35 years (OR, 2.28; 95% CI 1.67–3.13), pre-pregnancy underweight (OR, 2.36; 95% CI 1.60–3.47), pre-pregnancy overweight (OR, 1.67; 95% CI 1.09–2.56), and obesity (OR, 10.45; 95% CI, 3.91–27.87), nulliparity (OR, 0.58; 95% CI 0.41–0.82), pre-pregnancy diabetes (OR, 5.81; 95% CI 3.24–10.39), pre-pregnancy hypertension (OR, 2.79; 95% CI 1.44–5.41), and cervical incompetence (OR, 5.12; 95% CI 3.08–8.48) are independent risk factors for sPTB in twin pregnancies. The AUC of the training and validation set was 0.71 (95% CI 0.68–0.74) and 0.68 (95% CI 0.64–0.73), respectively. And then we integrated those risk factors to construct the nomogram.

**Conclusions:**

The nomogram developed for predicting the risk of sPTB in pregnant women with twins demonstrated good performance. The prediction nomogram serves as a practical tool by including all necessary predictors that are readily accessible to practitioners.

## Introduction

Spontaneous preterm birth (sPTB), denoting the autonomous delivery of a fetus before the culmination of 37 weeks of gestational progress, persists as a formidable challenge within the realm of obstetrics and gynecology, casting enduring shadows over the realms of maternal and neonatal well-being [1, 2]. Twin pregnancies, emblematic of the dual-fetal presence, introduce a dynamic interplay of genetic, environmental, and obstetric determinants, collectively shaping the landscape of preterm birth susceptibility [3, 4]. While the etiological underpinnings of sPTB in twins mirror those encountered in singleton pregnancies, the multifetal gestational context magnifies the intricate web of influences [5, 6]. Prevailing evidence suggested that the risk of preterm birth in a twin pregnancy is at least five times higher than a singleton pregnancy [7]. As of the present, approaches aimed at forestalling sPTB within twin pregnancies, encompassing interventions like vaginal progesterone, cervical pessary, and cervical cerclage, have encountered contentious debate or have been met with perceived limited efficacy [8, 9]. In order to meet the increasing demand for improved clinical practice guidelines, it is imperative to differentiate asymptomatic patients who have a higher risk of early sPTB from the overall population of women with twin pregnancies.

Prior investigations have solidified an association between sPTB in twin pregnancies and distinct clinical markers. These encompass factors like ethnic background, age, nulliparity, chorionicity, body mass index (BMI), tobacco consumption, past preterm delivery occurrences, cervical length, and the manifestation of funnelling [10–12]. A single predictor may exhibit limited efficacy in predicting sPTB, whereas a more accurate prediction can be achieved by integrating a predictive model that incorporates multiple predictors. Jun et al. devised and validated two-stage nomogram models, incorporating mid-gestation clinical characteristics, for predicting individual risk of preterm birth at < 34 weeks in twin pregnancies, demonstrating robust discrimination, calibration, and positive clinical benefit [13]. Subsequently, the researchers developed and validated a dynamic nomogram model to accurately predict the individual risk of sPTB before 32 weeks in twin pregnancies [14]. Predicting the risk of preterm birth based on pre-pregnancy or first trimester data holds greater significance for the implementation of interventions. However, the availability of models that demonstrate strong predictive performance in this context is limited and seldom reported. Therefore, there is still a need for further exploration and development of new models to enhance predictive efficacy.

In this study, we aimed to develop and validate prediction models of sPTB for twin pregnancies based on clinical features and laboratory tests to provide an accurate and comprehensive risk estimation as a diagnostic tool for clinical practice.

## Materials and methods

### Study design and data source

**Fig. 1:**
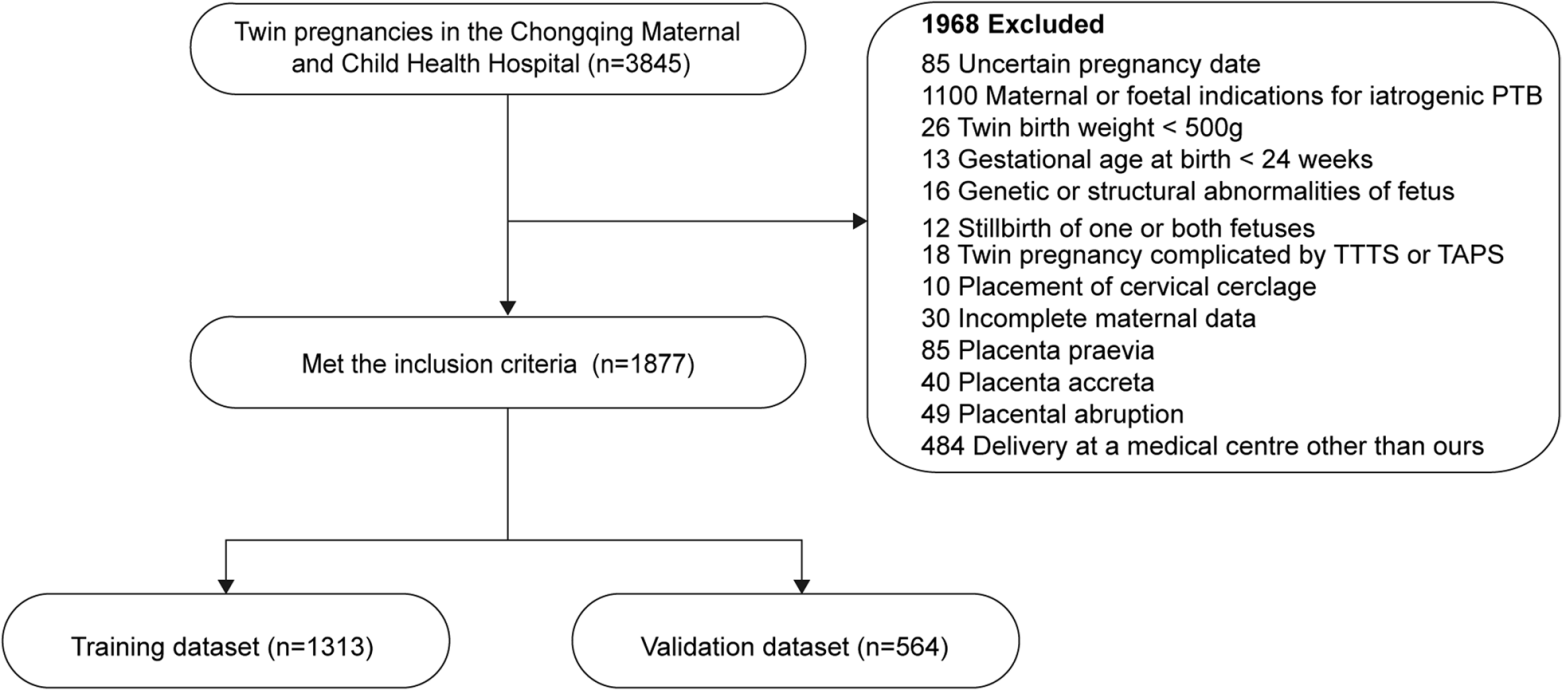
Flow chart of study design, inclusion and exclusion criteria and final study population. *PTB* preterm birth, *TTTS* twin-to-twin transfusion syndrome, *TAPS* twin anemia-polycythemia sequence

This study constitutes a retrospective cohort investigation involving twin pregnant women who attended Chongqing Maternal and Child Health Hospital before the 12th week of pregnancy, spanning the period from January 2017 to December 2022. Confirmation of twin pregnancy occurred through ultrasound examination around the 12th week. Our cohort comprises 3845 twin pregnant women. Alongside tracking the follow-up outcomes, we systematically documented demographic, clinical, and laboratory profiles for all participants. The data underwent meticulous collation using standardized data collection forms, subsequently subjected to thorough review by experienced medical professionals. The selection of patients for model development and validation is outlined in Fig. 1. The study was conducted in accordance with the guidelines of the Declaration of Helsinki. Ethical approval from the Ethics Committee of the Chongqing Maternal and Child Health Hospital stated that informed consent was not necessary due to the retrospective nature of the study and absence of patient interaction.

### Inclusion and exclusion criteria

The study utilized specific inclusion and exclusion criteria. Inclusion criteria encompassed women with twin pregnancies and deliveries occurring between 24 and 37 completed weeks of gestation, who were registered and planned to give birth at the Chongqing Maternal and Child Health Hospital, and had comprehensive medical records. The exclusion criteria encompassed cases with uncertain pregnancy date, maternal or fetal indications for iatrogenic preterm birth, twin birth weight < 500 g, gestational age at birth < 24 weeks, genetic or structural abnormalities of fetus, stillbirth of one or both fetuses, complications arising from monoamniotic or monochorionic twin pregnancy such as twin transfusion syndrome (TTTS) or twin anemia-polycythemia sequence (TAPS), cervical cerclage placement, incomplete maternal data, placenta previa, placenta accreta, placental abruption, or delivery at a different medical center. Consequently, a total of 1968 patients who met the exclusion criteria were excluded from the study, resulting in 1877 patients who met the inclusion criteria.

### Outcomes

For the purposes of this study, term delivery was defined as birth occurring from 37 completed weeks of gestation onwards, while preterm delivery was defined as birth occurring between 24 and 37 completed weeks of gestation [15]. The main outcome of our study was sPTB, which was specifically defined as preterm birth occurring after spontaneous contractions with intact membranes or preterm birth following the spontaneous rupture of membranes.

### Predictor variables

In this study, predictor variables were selected based on prior literature and their accessibility in clinical practice. These variables were extracted from demographic factors, clinical symptoms, and auxiliary or laboratory examinations. The baseline maternal information included the following variables: age (< 35 years and ≥ 35 years), pre-pregnancy BMI (underweight, normal, overweight, and obesity), nulliparity (yes and no), chorionicity (dichorionic, monochorionic, and monochorionic monoamniotic), mode of conception (natural pregnancy, in vitro fertilization, and ovulation induction), pre-pregnancy diabetes mellitus (yes and no), gestational diabetes mellitus (yes and no), pre-pregnancy hypertension (yes and no), hypertensive disorders of pregnancy (yes and no), scarred uterus (yes and no), infections (yes and no), cervical incompetence (yes and no), thyroid abnormalities (yes and no), anemia (no, mild, moderate, or severe), hypoproteinemia (yes and no), and thrombocytopenia (yes and no).

Pre-pregnancy BMI was calculated as pre-pregnancy weight (kg) divided by height (m) squared [16]. The categories for pre-pregnancy BMI were as follows: underweight (< 18.5 kg/m²), normal (18.5–24.9 kg/m²), overweight (25–29.9 kg/m²), and obesity (≥ 30 kg/m²) [17]. Pre-pregnancy diabetes of pregnant women was defined as type 1 or type 2 diabetes diagnosed before pregnancy [18]. Gestational diabetes mellitus is defined as a diagnosis of hyperglycaemia during pregnancy, in a woman without pre-existing diabetes mellitus [19]. Pre-pregnancy hypertension was defined as hypertension predating the pregnancy or diagnosed before the 20th week of pregnancy [20]. Hypertensive disorders of pregnancy included preeclampsia, eclampsia, and chronic hypertension with superimposed preeclampsia. Infections considered were gonorrhea, syphilis, chlamydia, hepatitis B, hepatitis C, and Group B streptococci (GBS). The definition of cervical insufficiency includes three criteria: painless cervical dilation of 1 cm or more, exposed or bulging fetal membranes without uterine contractions as visually determined using a sterile speculum, or cervical length equal to or less than 25 mm before 24 weeks of gestation [21]. Thyroid dysfunction abnormalities was identified based on self-reported physician diagnosis of hyperthyroidism or hypothyroidism, or the use of antithyroid drugs. The severity of anemia was categorized as follows: hemoglobin level between 11 and 11.9 g/dL was considered mild anemia, 8–10.9 g/dL moderate anemia, and < 8 g/dL severe anemia [22]. Hypoproteinemia was defined as serum albumin levels < 30 g/L, and thrombocytopenia was defined as a platelet count < 100,000/µL [23, 24].

### Sample size

In determining the sample size for our predictive model, our study employed a data-driven approach, utilizing existing datasets to establish the requisite sample size. Drawing from Kendall’s prescription, advocating that studies elucidating factors of influence require a sample size encompassing 5 to 10 times the number of variables, we navigated a total of 16 variables within this study [25]. Computed through the criterion of a sample size amplification of 10 times the number of variables, our analysis mandated no fewer than 160 patients for comprehensive coverage.

### Missing value processing

In the process of constructing our predictive model, a total of 1877 participants were included in the final sample from our comprehensive dataset, which comprised 16 variables. During the data preprocessing stage, it was noted that some variables contained missing values. Specifically, thyroid dysfunction abnormalities had 10 missing values, cervical incompetence had 12 missing values, and thrombocytopenia had 5 missing values. To address the issue of missing data and ensure the integrity of our analysis, we employed a multiple imputation technique [26]. For our study, we conducted 10 imputation iterations to ensure stability and convergence of the imputation process.

### Statistical analysis

Clinical and laboratory data were obtained from the medical records of the hospital. The population was then randomly divided into a training set and a validation set in a ratio of 70–30%. Categorical variables were presented as numbers and percentages [n (%)], and the groups were compared using χ^2^ tests or Fisher’s exact tests. Univariate and multivariate logistic regression analyses were performed to identify correlations between clinical variables and sPTB. Before including variables in the regression models, a collinearity diagnostic test was conducted on all explanatory variables to assess collinearity. Collinearity was assessed using variance inflation factors (VIF), and a VIF less than 5 indicated no collinearity among the variables. The logistic regression method was then used to construct the final model. The discriminatory ability of the models was assessed using the area under the receiver operating characteristic (ROC) curve, and the calibration of the nomogram model was assessed using calibration curves. Sensitivity, specificity and accuracy were determined from the optimal threshold using the Youden index. Clinical utility was assessed via decision curve analysis (DCA). All statistical analyses were performed using SPSS (version 24.0, IBM Corp., Armonk, NY, USA) and the R statistical computing language (version 3.4.3). All statistical tests were two-tailed, and statistical significance was considered as *P* < 0.05.

## Results

### Characteristics of participants

A total of 3848 women with twin pregnancies were screened and 1877 patients were finally included in this study according to the inclusion and exclusion criteria (Fig. 1). Of these women, 555 (29.6%) had sPTB, 329 (17.5%) were ≥ 35 years, 1478 (78.7%) had normal pre-pregnancy BMI, 1521 (81.0%) were nulliparity, 1627 (86.7%) were dichorionic diamniotic pregnancy, 528 (28.1%) were spontaneous conception, 86 (4.6%) had pre-pregnancy diabetes, 487 (25.9%) had gestation diabetes, 60 (3.2%) had pre-pregnancy hypertension, 234 (12.5%) had gestation hypertention, 128 (6.8%) had scarred uterus, 164 (8.7%) had infections, 119 (6.3%) had cervical incompetence, 198 (10.5%) had thyroid abnormalities, 1449 (77.2%) had normal hemoglobin, 168 (9.0%) had hypoproteinemia, and 102 (5.4%) had thrombocytopenia. Subsequently, the included women were randomly divided into a training and validation set according to the sample size ratio of 7:3 (training set: 1313; validation set: 564). No significant differences were observed in maternal demographic and clinical characteristics between the training and validation sets (*P* > 0.05), underscoring the similarity in features between the training and internal validation sets (Table 1). This equivalence suggests that subsequent internal validation is poised to be representative.

### Differences in women with and without sPTB in the training set

Table 2 shows the differences in women with and without sPTB in the training set. The results indicated that there were differences in age (*P* < 0.001), pre-pregnancy BMI (*P* < 0.001), nulliparity (*P* = 0.045), pre-pregnancy diabetes (*P* < 0.001), pre-pregnancy hypertension (*P* = 0.001), and cervical incompetence (*P* < 0.001), between women with and without sPTB.

**Table 1:** Characteristics of the women with twin pregnancies in the training and validation sets

Characteristics	Training set (n = 1313)	Validation set (n = 564)	*P* value
Age (years), n (%)			0.857
< 35	1081 (82.3)	467 (82.8)	
≥ 35	232 (17.7)	97 (17.2)	
Pre-pregnancy BMI (kg/m^2^), n (%)			0.575
Normal	1035 (78.8)	443 (78.5)	
Underweight	136 (10.4)	63 (11.2)	
Overweight	120 (9.14)	53 (9.40)	
Obesity	22 (1.68)	5 (0.89)	
Nulliparity, n (%)			0.277
No	258 (19.6)	98 (17.4)	
Yes	1055 (80.4)	466 (82.6)	
Chorionicity, n (%)			0.460
Dichorionic diamniotic	1131 (86.1)	496 (87.9)	
Monochorionic diamniotic	179 (13.6)	68 (12.1)	
Monochorionic monoamniotic	3 (0.23)	0 (0.00)	
Conception modalities, n (%)			0.106
Spontaneous conception	386 (29.4)	142 (25.2)	
IVF	909 (69.2)	417 (73.9)	
Ovulation induction	18 (1.37)	5 (0.89)	
Pre-pregnancy diabetes, n (%)			0.935
No	1252 (95.4)	539 (95.6)	
Yes	61 (4.65)	25 (4.43)	
Gestation diabetes, n (%)			0.310
No	963 (73.3)	427 (75.7)	
Yes	350 (26.7)	137 (24.3)	
Pre-pregnancy hypertension, n (%)			0.893
No	1272 (96.9)	545 (96.6)	
Yes	41 (3.12)	19 (3.37)	
Hypertensive disorders of pregnancy, n (%)			0.739
No	1152 (87.7)	491 (87.1)	
Yes	161 (12.3)	73 (12.9)	
Scarred uterus, n (%)			0.554
No	1220 (92.9)	529 (93.8)	
Yes	93 (7.08)	35 (6.21)	
Infections, n (%)			0.620
No	1195 (91.0)	518 (91.8)	
Yes	118 (8.99)	46 (8.16)	
Cervical incompetence, n (%)			0.110
No	1238 (94.3)	520 (92.2)	
Yes	75 (5.71)	44 (7.80)	
Thyroid abnormalities, n (%)			0.869
No	1176 (89.6)	503 (89.2)	
Yes	137 (10.4)	61 (10.8)	
Anemia, n (%)			0.457
Normal	1002 (76.3)	447 (79.3)	
Mild	184 (14.0)	64 (11.3)	
Moderate	100 (7.62)	42 (7.45)	
Severe	27 (2.06)	11 (1.95)	
Hypoproteinemia, n (%)			0.727
No	1193 (90.9)	516 (91.5)	
Yes	120 (9.14)	48 (8.51)	
Thrombocytopenia, n (%)			0.112
No	1234 (94.0)	541 (95.9)	
Yes	79 (6.02)	23 (4.08)	

*BMI* body mass index, *IVF* in vitro fertilization-embryo transfer

**Table 2:** Characteristics of twin pregnancy women with and without sPTB in the training set

Characteristics	Control (n = 928)	sPTB (n = 385)	*P*
Age (years), n (%)			< 0.001
< 35	796 (85.8)	285 (74.0)	
≥ 35	132 (14.2)	100 (26.0)	
Pre-pregnancy BMI (kg/m^2^), n (%)			< 0.001
Normal	769 (82.9)	266 (69.1)	
Underweight	77 (8.30)	59 (15.3)	
Overweight	76 (8.19)	44 (11.4)	
Obesity	6 (0.65)	16 (4.16)	
Nulliparity, n (%)			0.045
No	196 (21.1)	62 (16.1)	
Yes	732 (78.9)	323 (83.9)	
Chorionicity, n (%)			0.114
Dichorionic diamniotic	810 (87.3)	321 (83.4)	
Monochorionic diamniotic	116 (12.5)	63 (16.4)	
Monochorionic monoamniotic	2 (0.22)	1 (0.26)	
Conception modalities, n (%)			0.308
Spontaneous conception	264 (28.4)	122 (31.7)	
IVF	653 (70.4)	256 (66.5)	
Ovulation induction	11 (1.19)	7 (1.82)	
Pre-pregnancy diabetes, n (%)			< 0.001
No	909 (98.0)	343 (89.1)	
Yes	19 (2.05)	42 (10.9)	
Gestation diabetes, n (%)			0.694
No	684 (73.7)	279 (72.5)	
Yes	244 (26.3)	106 (27.5)	
Pre-pregnancy hypertension, n (%)			0.001
No	909 (98.0)	363 (94.3)	
Yes	19 (2.05)	22 (5.71)	
Hypertensive disorders of pregnancy, n (%)			0.154
No	806 (86.9)	346 (89.9)	
Yes	122 (13.1)	39 (10.1)	
Scarred uterus, n (%)			0.216
No	868 (93.5)	352 (91.4)	
Yes	60 (6.47)	33 (8.57)	
Infections, n (%)			0.144
No	852 (91.8)	343 (89.1)	
Yes	76 (8.19)	42 (10.9)	
Cervical incompetence, n (%)			< 0.001
No	900 (97.0)	338 (87.8)	
Yes	28 (3.02)	47 (12.2)	
Thyroid abnormalities, n (%)			0.894
No	830 (89.4)	346 (89.9)	
Yes	98 (10.6)	39 (10.1)	
Anemia, n (%)			0.069
Normal	726 (78.2)	276 (71.7)	
Mild	119 (12.8)	65 (16.9)	
Moderate	67 (7.22)	33 (8.57)	
Severe	16 (1.72)	11 (2.86)	
Hypoproteinemia, n (%)			0.364
No	848 (91.4)	345 (89.6)	
Yes	80 (8.62)	40 (10.4)	
Thrombocytopenia, n (%)			0.234
No	867 (93.4)	367 (95.3)	
Yes	61 (6.57)	18 (4.68)	

*sPTB* spontaneous preterm birth, *BMI *body mass index, *IVF* in vitro fertilization-embryo transfer

### Univariate and multivariate regression analysis of risk factors for sPTB in the training set

Table 3 provides an insightful presentation of both univariate and multivariate analyses pertaining to the factors associated with sPTB in the training set. Univariate analysis discerned significant associations (*P* < 0.05) between sPTB and certain variables, including age ≥ 35 years, pre-pregnancy overweight, underweight and obesity, nulliparity, pre-pregnancy diabetes, pre-pregnancy hypertension, and cervical incompetence. Subsequently, the singularly significant factors identified in the univariate analysis were subjected to multivariate logistic regression. The multivariate analysis established that age ≥ 35 years (OR, 2.28; 95% CI 1.67–3.13), pre-pregnancy underweight (OR, 2.36; 95% CI 1.60–3.47), pre-pregnancy overweight (OR, 1.67; 95% CI 1.09–2.56), and obesity (OR, 10.45; 95% CI 3.91–27.87), nulliparity (OR, 0.58; 95% CI 0.41–0.82), pre-pregnancy diabetes (OR, 5.81; 95% CI 3.24–10.39), pre-pregnancy hypertension (OR, 2.79; 95% CI 1.44–5.41), and cervical incompetence (OR, 5.12; 95% CI 3.08–8.48) remained significantly associated with an elevated risk of sPTB.

**Table 3:** Univariate and multivariate analyses of factors associated with sPTB in twin pregnancies within the training set

Characteristics	Univariate		Multivariate	
	OR (95%CI)	*P*	OR (95%CI)	*P*
Age (years), n (%)				
< 35	Ref.		Ref.	
≥ 35	2.11 (1.57–2.83)	< 0.001	2.28 (1.67–3.13)	< 0.001
Pre-pregnancy BMI (kg/m^2^), n (%)				
Normal	Ref.		Ref.	
Underweight	2.22 (1.54–3.20)	< 0.001	2.36 (1.60–3.47)	< 0.001
Overweight	1.67 (1.13–2.49)	0.010	1.67 (1.09–2.56)	0.018
Obesity	7.71 (2.99–19.91)	< 0.001	10.45 (3.91–27.87)	< 0.001
Nulliparity, n (%)				
Yes	Ref.		Ref.	
No	0.72 (0.52–0.98)	0.038	0.58 (0.41–0.82)	0.002
Chorionicity, n (%)				
Dichorionic diamniotic	Ref.		–	–
Monochorionic diamniotic	1.37 (0.98–1.91)	0.064	–	–
Monochorionic monoamniotic	1.26 (0.11–13.96)	0.850	–	–
Conception modalities, n (%)				–
IVF	Ref.		–	–
Spontaneous conception	1.18 (0.91–1.53)	0.213	–	–
Ovulation induction	1.62 (0.62–4.23)	0.322	–	–
Pre-pregnancy diabetes, n (%)				
No	Ref.		Ref.	
Yes	5.85 (3.36–10.21)	< 0.001	5.81 (3.24–10.39)	< 0.001
Gestation diabetes, n (%)				
No	Ref.		–	–
Yes	1.07 (0.81–1.39)	0.644	–	–
Pre-pregnancy hypertension, n (%)				
No	Ref.		Ref.	
Yes	2.89 (1.55–5.42)	< 0.001	2.79 (1.44–5.41)	0.002
Hypertensive disorders of pregnancy, n (%)				
No	Ref.		–	–
Yes	0.74 (0.51–1.09)	0.130	–	–
Scarred uterus, n (%)				
No	Ref.		–	–
Yes	1.36 (0.87–2.11)	0.177	–	–
Infections, n (%)				–
No	Ref.		–	
Yes	1.37 (0.92–2.04)	0.118	–	
Cervical incompetence, n (%)				
No	Ref.		Ref.	
Yes	4.47 (2.75–7.25)	< 0.001	5.12 (3.08–8.48)	< 0.001
Thyroid abnormalities, n (%)				
No	Ref.		–	–
Yes	0.95 (0.64–1.41)	0.816	–	–
Anemia, n (%)				
Normal	Ref.		–	–
Mild	1.43 (1.03-2.00)	0.693	–	–
Moderate	1.29 (0.83–2.01)	0.248	–	–
Severe	1.80 (0.82–3.94)	0.454	–	–
Hypoproteinemia, n (%)				
No	Ref.		–	–
Yes	1.23 (0.82–1.83)	0.311	–	–
Thrombocytopenia, n (%)				–
No	Ref.		–	–
Yes	0.69 (0.41–1.19)	0.190	–	–

*sPTB* spontaneous preterm birth, *BMI* body mass index, *IVF* in vitro fertilization-embryo transfer

### Model performance and validation

**Fig. 2:**
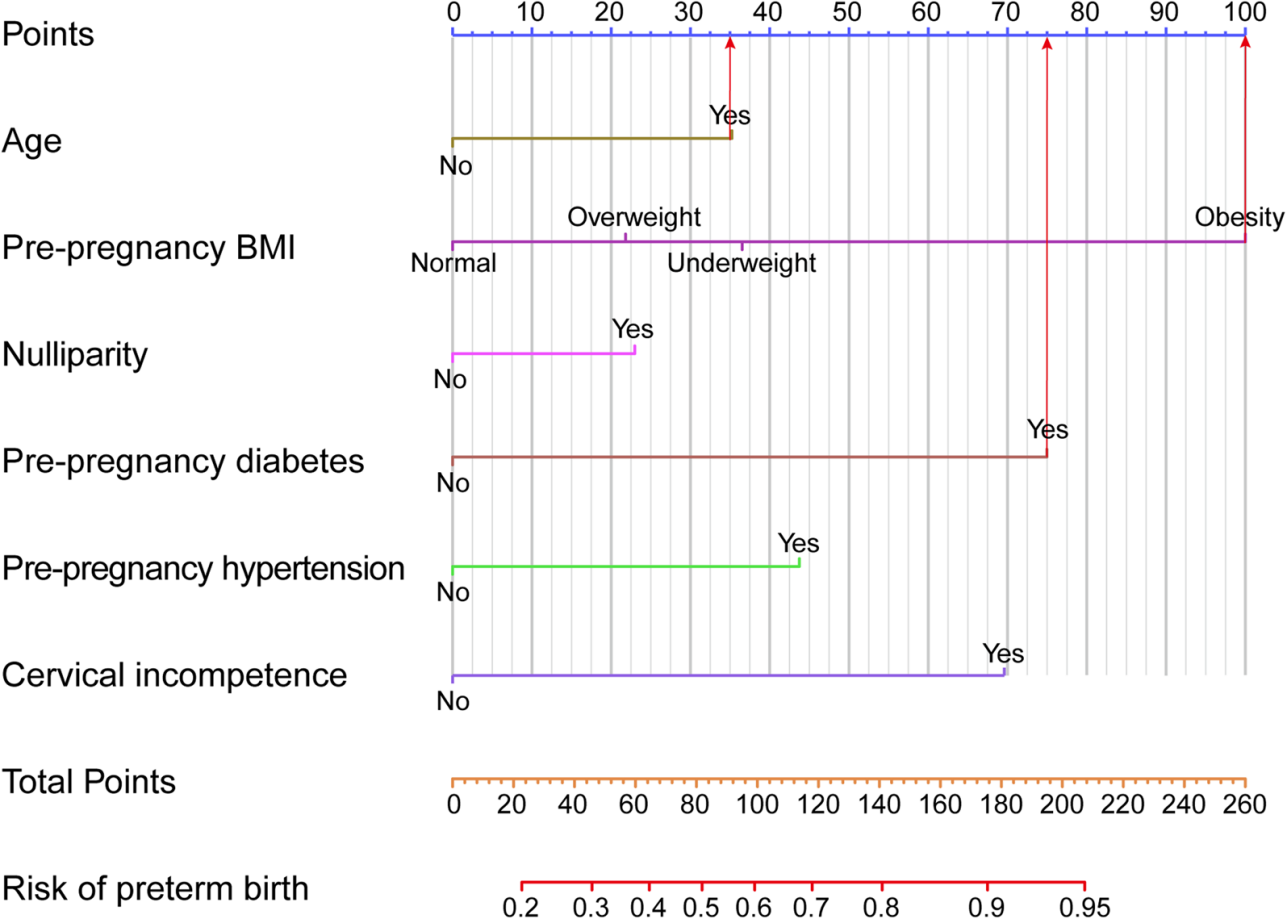
Nomogram for predicting the occurrence of sPTB in twin pregnancies women. In order to determine the likelihood of sPTB in twin pregnancies, the assignment of points for each variable is based on the corresponding value along the “point” axis, and these points are subsequently plotted on the axis representing the total points. The cumulative risk of sPTB in twin pregnancies is then determined by the total points obtained

**Fig. 3:**
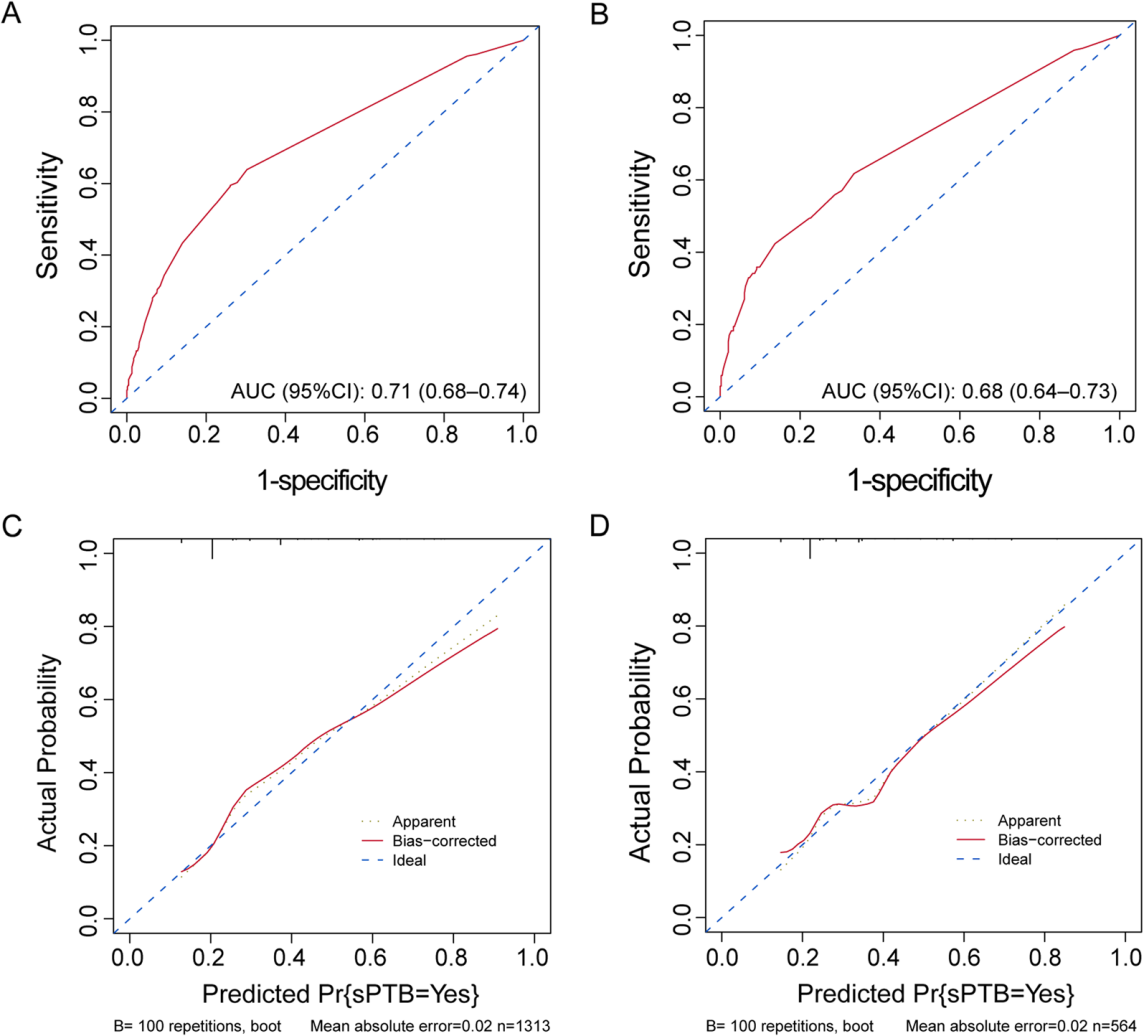
The assessment of discrimination and calibration of the predictive models. ** A** ROC curve of the training set. **B** ROC curve of the validation set. **C** Calibration curves of the training set. **D** Calibration curves of the validation set

**Fig. 4:**
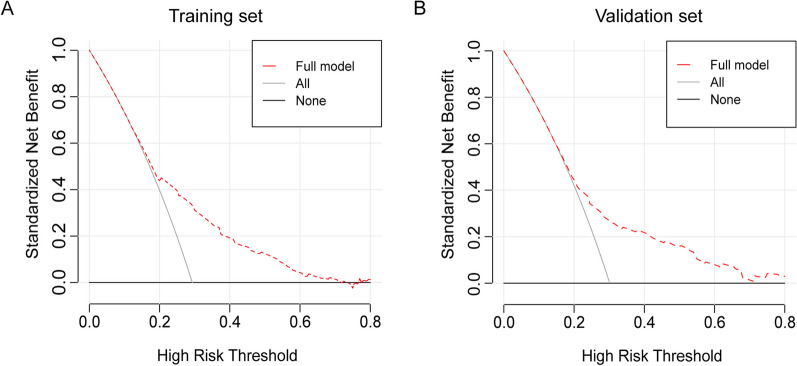
Decision curve analysis (DCA) of the nomogram. ** A** DCA of the nomogram for sPTB prediction of twin pregnancy in the training set. **B** DCA of the nomogram for sPTB prediction of twin pregnancy in the validation set

Variables such as age, pre-pregnancy BMI, nulliparity, pre-pregnancy diabetes, pre-pregnancy hypertension, and cervical incompetence were included in the development of a prediction model. Table 4 presents the performance of the model in the training and validation sets. Using the Youden index, the cut-off point was determined to be 0.23. The AUC of the model was 0.71 (95% CI 0.68–0.74) in the training set and 0.68 (95% CI 0.64–0.73) in the validation set. The accuracy of the model was 0.74 (95% CI 0.72–0.77) in the training set and 0.75 (95% CI 0.71–0.78) in the validation set. Additionally, Fig. 2 shows the nomogram used for predicting the occurrence of sPTB in pregnant women. Each point on the nomogram corresponds to an intersection of the variable’s vertical line with the point axis, and the total risk score is calculated by summing the points for each variable. The probability of twin sPTB can be determined by reading the total point axis. For instance, a 38-year-old (35 points) pregnant woman with diabetes (75 points) and a BMI of 30 (100 points) would accumulate a total of 210 points, corresponding to a risk probability of > 95%. The Harrell’s concordance index value of the nomogram model in the training set was 0.71 (95% CI 0.68–0.74). When applied to the validation set, the Harrell’s concordance index value was 0.68 (95% CI 0.64–0.73) (Fig. 3A, B). The calibration curves demonstrate good agreement between the predicted probabilities from the nomogram and the actual probabilities in both the training and validation sets (Fig. 3C, D). The results of the DCA showed that the nomogram had good accuracy in predicting the sPTB of twin pregnancy (Fig. 4A, B).

**Table 4:** Performances of the model in the training set and validation set

Parameters	Training set	Validation set
AUC (95%CI)	0.71 (0.68–0.74)	0.68 (0.64–0.73)
Accuracy (95%CI)	0.74 (0.72–0.77)	0.75 (0.71–0.78)
Specificity (95%CI)	0.70 (0.67–0.80)	0.86 (0.64–0.93)
Sensitivity (95%CI)	0.64 (0.54–0.68)	0.42 (0.33–0.69)
PPV (95%CI)	0.64 (0.57–0.72)	0.66 (0.55–0.76)
NPV (95%CI)	0.76 (0.73–0.78)	0.76 (0.72–0.80)

*AUC* area under the curve, *PPV* positive predictive value, *NPV* negative predictive value, *CI* confidence interval

## Discussion

### Principal findings

In our investigation, we showed the integration of maternal demographic traits and cervical incompetence at mid-gestation into nomograms for estimating individualized risk of sPTB in twin pregnancies. Following two rounds of screening, the ultimate model integrated five predictors: age, pre-pregnancy BMI, nulliparity, pre-pregnancy diabetes, pre-pregnancy hypertension, and cervical incompetence. The resultant model was depicted in the format of a nomogram. In general, the risk model exhibited a discernible level of calibration and effective discrimination in the context of this study.

### Strengths and interpretation

Although the mechanism underlying the onset of premature labor in twin pregnancies may differ from that in singleton pregnancies, research has shown that demographic factors contribute to the etiology of sPTB [27]. In our investigation, we identified that risk factors including age, pre-pregnancy BMI, nulliparity, pre-pregnancy diabetes, pre-pregnancy hypertension, and cervical incompetence are correlated with preterm delivery. Research indicates a 36% increase in preterm birth rates in Canada from 1990 to 1996 associated with rising maternal age, although findings on the specific impact of high maternal age on preterm birth remain inconsistent [28]. Studies, including a Danish cohort, highlight a U-shaped relationship between maternal age and preterm birth risk, with the lowest risk observed between ages 24–30 [29]. Consistent with previous research [30], this study affirms that maternal age over 35 is an independent risk factor for sPTB in women with twin pregnancies. Maternal BMI is intricately linked to preterm birth risk, with both low and high BMI levels associated with increased likelihoods of sPTB. Lower BMI is linked to a 1.3-fold higher risk of sPTB, potentially due to chronic malnutrition and nutrient deficiencies impacting fetal development [30]. Meanwhile, women with a BMI over 40.7 face a 3.0-fold higher risk of sPTB [31]. This study, in concordance with others, underscores that underweight, overweight, and obesity independently elevate the risk of sPTB in twin pregnancies. Elevated parity has been a subject of diverse findings in relation to sPTB, with some studies indicating an increased likelihood of adverse outcomes, while others argue that under favorable socio-economic and healthcare conditions, it may not be a significant risk factor [32–35]. Building on this, Bouchra Koullali et al. identified an independent association between nulliparity and sPTB in various gestational weeks [36]. Additionally, our study introduces a novel perspective by examining the impact of diabetes mellitus and chronic hypertension on sPTB risk in twin pregnancies, revealing these conditions as additional risk factors [37–42]. Understanding these relationships contributes to developing effective preventive strategies for sPTB in diverse populations.

Transvaginal sonography effectively predicts preterm birth, particularly in twin pregnancies, highlighting the significance of cervical incompetence, especially with cervical shortening in the late second trimester [43–45]. A 2010 systematic review found that a single transvaginal ultrasound measurement of cervical length between 20 and 24 weeks reliably predicted sPTB at < 28, < 32, and < 34 weeks in asymptomatic women with twin pregnancies [46]. Additional risk factors for sPTB include cervical funneling and exposed fetal membranes without uterine contractions [47, 48]. However, some studies question the clinical utility of cervical funneling for predicting preterm birth in both asymptomatic and symptomatic women with twin pregnancies [49, 50]. Our study classified cervical incompetence indicators as a short cervix, cervical funneling, cervical dilatation, and fetal membrane bulging, finding cervical insufficiency to be a significant risk factor for sPTB. Yet, evidence supporting routine monitoring of cervical function in women with twin pregnancies is lacking, raising questions about its health benefits outweighing the additional workload. Concerns include delayed detection of cervix length shortening in the late second or early third trimester, potentially impacting the timing of antenatal corticosteroid therapy and in utero transfers. Hence, timely and accurate assessments for women at risk of preterm labor are crucial.

Nomograms offer a practical graphical representation of intricate logistic regression models, enabling the derivation of individualized scores by summing up points for each variable. While predictive models for sPTB are largely tailored for singleton pregnancies, twin pregnancies have seen fewer dedicated systems [51]. This study addresses this gap by introducing and validating prediction models, serving as a user-friendly online tool utilizing routine clinical data from primary care units. The model demonstrates satisfactory performance in assessing an individual’s comprehensive risk of sPTB, displaying favorable discrimination and calibration. However, our findings highlight lower sensitivity, potentially linked to the absence of crucial clinical information or imbalanced variables among different groups. The model might exhibit a preference for predicting categories with more samples, resulting in lower sensitivity for less populated categories. Despite these considerations, our models contribute to clinical benefits. Decision curve analysis indicates proximity to the net benefits at respective threshold probabilities. Notably, a model with higher specificity is deemed valuable for screening. This underscores the significance of promoting prediction models as a cost-effective strategy.

### Comparison with previous prediction models

Our new models are well calibrated when applied to a separate validation cohort and have high levels of discrimination. Currently, there is a limited amount of research on predictive models for sPTB in twin pregnancies. Zhang Jun et al. have developed a dynamic model to predict the risk of spontaneous preterm birth in twin pregnancies before 32 weeks of gestation. Their model demonstrated a sensitivity of 80.00%, specificity of 88.17%, positive predictive value of 50.33%, and negative predictive value of 96.71% [14]. In comparison, our model not only incorporates additional variables but also achieves discrimination in predicting sPTB. However, when compared to predictive models for sPTB in singleton pregnancies, our model exhibits slightly inferior performance in terms of sensitivity and specificity [52, 53].

### Limitations of this study

However, there are several limitations in our study that need to be addressed. Firstly, the study was conducted in a single center, which lacks external validation from other centers, thus introducing bias. Secondly, being a retrospective study, it is not possible to completely eliminate potential biases. Lastly, our analysis may not have encompassed all possible factors related to sPTB. For instance, important risk factors like smoking, previous history of preterm birth, and genital tract infections may have an impact on sPTB, but data regarding these factors were not included in our study.

## Conclusion

In summary, this study examined the risk factors associated with sPTB in twin pregnancies. The results indicate that advanced maternal age, pre-pregnancy underweight or overweight, nulliparity, pre-pregnancy hypertension and diabetes, and cervical insufficiency are significant factors contributing to sPTB. Furthermore, a nomogram was utilized to visualize the predictive model, demonstrating satisfactory discrimination and calibration capabilities. Nevertheless, further prospective research is essential to investigate additional risk factors and validate the efficacy of our prediction tool.

## Data Availability

The datasets used and/or analysed during the current study are available from the corresponding author on reasonable request.

## Abbreviations

sPTB: Spontaneous preterm birth
OR: Odds ratio
CI: Confidence interval
AUC: Area under the receiver operating characteristic curve
BMI: Body mass index
TTTS: Twin transfusion syndrome
TAPS: Twin anemia-polycythemia sequence
VIF: Variance inflation factors
DCA: Decision curve analysis
